# *Bifidobacterium pseudonumeratum* W112 alleviated depressive and liver injury symptoms induced by chronic unpredictable mild stress via gut-liver-brain axis

**DOI:** 10.3389/fnut.2024.1421007

**Published:** 2024-08-19

**Authors:** Jingqi Zhao, Jiahu Yuan, Yihua Zhang, Langni Deng, Yajing Pan, Xiaojia Bai, Longgang Jia, Yanping Wang, Weitao Geng

**Affiliations:** ^1^Food Science and Engineering, Tianjin University of Science and Technology, Tianjin, China; ^2^Key Laboratory of Traditional Chinese Medicine Lifeomics and Innovative Drug Research and Development, Shanxi University of Chinese Medicine, Taiyuan, China

**Keywords:** *Bifidobacterium pseudocatenulatum*, depression, gut microbiota, liver injury, chronic unpredictable mild stress (CUMS)

## Abstract

**Introduction:**

Several studies indicated that depression is associated with liver injury. The role of probiotics in alleviating depression is focused on improving the abnormalities of the central nervous system through the gut-brain axis, while the effect on liver injury is still unclear. The aim of this study was to elucidate the potential link between the antidepressant effect of a potential probiotic strain *Bifidobacterium pseudocatenulatum* W112 and its effect on alleviating liver injury.

**Methods:**

The 4-week-old Kunming mice were exposed to chronic stress for 4 weeks to establish a depression model.

**Results:**

The depression-like behavior and related biomakers in chronic unpredictable mild stress (CUMS) mice were altered by supplemented with W112 for 2 weeks. Meanwhile, the modulation effect of W112 the gut microbiota in CUMS mice also result in an increase in the abundance of beneficial bacteria and a decrease in the abundance of harmful bacteria. Significantly, liver injury was observed in CUMS model mice. W112 improved liver injury by reducing AST/ALT in serum. Quantitative PCR results indicated that the mechanism of action of W112 in ameliorating liver injury was that the altered gut microbiota affected hepatic phospholipid metabolism and bile acid metabolism.

**Discussion:**

In short, W112 could significantly improve the depressive and liver injury symptoms caused by CUMS. The gut-liver-brain axis is a potential connecting pathway between the antidepressant effects of W112 and its alleviation of liver injury.

## Highlights

- This study elucidates that the gut liver brain axis is a potential connecting pathway between the antidepressant effect of W112 and its reduction of liver injury.
- *Bifidobacterium pseudocatenulatum* W112 isolated from healthy infant feces can alleviate depression caused by CUMS by optimizing the composition of gut microbiota.
- W112 can promote the normalization of neuroendocrine activity by regulating the content of relevant biomarkers in CUMS mice, which indicated that W112 has an antidepressant function.
- W112 could alleviate CUMS induced liver injury by regulating phospholipid metabolism and bile acid metabolism.

## 1 Introduction

Depression, which is characterized by continuous and prolonged gloom, is one of the most common mental diseases. According to the report from the World Health Organization (WHO), there are more than 300 million people with depression worldwide. It is expected that depression will become the first major disease threatening human health in 2030 (1). Medication is an effective method for depression. However, most drugs have terrible side effects after being used for a long time (2, 3). Therefore, it is necessary to develop more alternative treatment to avoid the side effects of anti-depressant drugs and alleviate the depressive symptoms.

As early as 1910, Dr. J George Porter Phillips first put forward the idea that lactic acid bacteria could improve mental health (4). With the proposal and development of microorganism-gut-brain axis theory, it is realized that microorganism and brain can exchange regulatory signals in bidirectional communication pattern (5). Therefore, it is widely concerned to explore the relationship between probiotics and depression (6). It has been confirmed that a variety of probiotics, including *Bifidobacterium, Lactobacillus, Enterococcus*, etc., could change the mood and cognition by acting on the enteric nervous system and immune system (7, 8). However, there are few researches about the effect of probiotics on other organ injury caused by depression (9). The liver, which has the functions of detoxification, is an essential metabolic organ in the body. Previous studies have shown that stress can induce liver injury in a direct or indirect manner. Among them, the excessive inflow of lipopolysaccharide (LPS), high levels of stress hormones, increased sympathetic nervous system activity and other factors are considered to play an important role in liver injury (10). The Bifidobacterium was reported to protect liver from injury by modulating gut microbiota to reduce the excessive of intestinal derived LPS into the liver (11). Therefore, it is essential to strengthen the study on the link between the antidepressant effect of the potential probiotic and its alleviation of liver injury.

The novel potential probiotic strain *Bifidobacterium pseudocatenulatum* W112 (W112) was isolated from healthy infant feces in our previous work. Herein, the effect of W112 on CUMS mice was systematically investigated by behavior experiment, brain neurophysiological alterations. Meanwhile, the effect of W112 on liver injury caused by CUMS was also investigated by detecting the related-biomarkers and the expression of metabolism-related gene in liver. Furthermore, the mechanism and potential link of antidepressant and hepatic injury reduction of W112 was analyzed by gut microbiota sequencing. This study provides a new perspective for understanding the probiotics in alleviating depression.

## 2 Materials and methods

### 2.1 The probiotic strain and animal

*Bifidobacterium pseudocatenulatum* W112 (W112) was cultured in tryptone phytone yeast (TPY) medium at 37°C under anaerobic conditions. 4-weeks-old, specific pathogen free (SPF) Kunming male mice were purchased from Experimental Animal Medical Research Center of Changchun Academy of Military Medical Sciences. Mice were reared at relative humidity of 55 ± 5%, 2°C and 12/12 h light/dark cycle. All of the animal experiments in this study were carried out in accordance with the U.S. National Institutes of Health and approved by the Institutional Animal Care and Use Committee at the Tianjin University of Science and Technology (approve number. TUST20201206).

### 2.2 Establishment of CUMS model and intervention protocols

The method for CUMS model refers to the report by Willner (12). Briefly, mice in the CUMS and intervention groups were exposed to seven random discontinuous stressors every day. The stressors included cold water swimming (4°C, 4 min), food deprivation (24 h), water deprivation (24 h), clip tail (1 min), perversion day and night (24 h), tilted cage (45°C, 7 h), and soiled cage (24 h).

**Figure 1:**
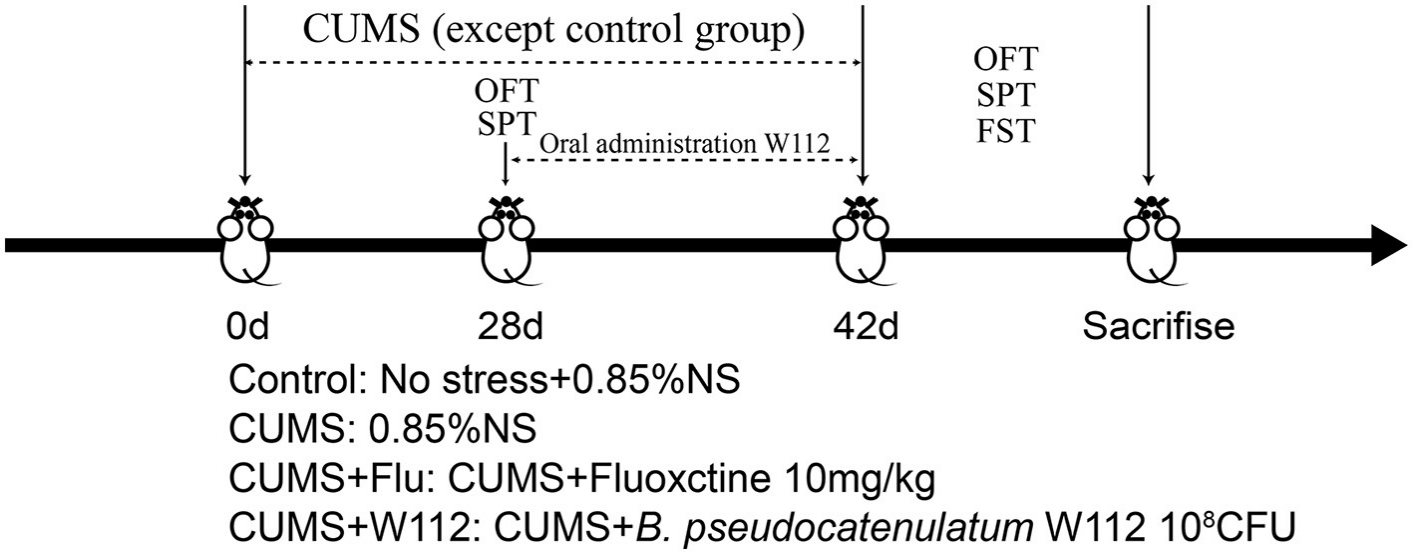
Administration scheme of *Bifidobacterium pseudocatenulatum* W112.

After acclimatizing to the animal room for 3 days, 40 male mice were randomly divided into 4 groups: control group, CUMS group, Fluoxetine hydrochloride (Flu; Beijing Huaxia Ocean Technology Co., Ltd. Beijing, China) intervention group (positive control group, CUMS+Flu), W112 intervention group (CUMS+W112). Mice in the control and CUMS groups were orally administered 0.2 mL 0.85% sterile saline. Mice in the CUMS+Flu group were orally administered with Flu (10 mg/kg). Mice in the CUMS+W112 group were orally administered with 10^8^ colony-forming unit (CFU) of strain W112. The experimental design is shown in Figure 1. During the experiment, the body weight of each mouse was recorded every 7 days. The fresh blood, feces and tissues including hypothalamus and livers were obtained for later pathological detection after the mice were sacrificed.

### 2.3 Behavioral test

Depressive symptoms of mice were directly assessed by behavioral test, including sucrose preference test (SPT), open field test (OFT), and forced swimming test (FST) on the 28th day and 42nd day of experiment (the detail protocol see Supplementary material).

### 2.4 Detection of biochemical markers

The serum was separated from the collected blood by centrifugation at 1359 g for 10 min (Sigma 3-18 K, GER). The contents of Tryptophan (Trp), kynurenine (KYN), kynurenic acid (KYNA), corticosterone (CORT), adrenergic hormone (ACTH) in the serum were measured by the corresponding enzyme-linked immuno sorbent assay (ELISA) kits (Tianjin LAB Technology Co., Ltd., Tianjin, China). The contents of Trp, 5-hydroxytryptamine (5-HT), 5-hydroxyindoleacetic acid (5-HIAA), corticotrophin releasing hormone (CRH) in the hypothalamus were measured by corresponding ELISA kits (Tianjin LAB Technology Co., Ltd., Tianjin, China). The contents of alanine aminotransferase (ALT) and aspartate aminotransferase (AST) in the serum were measured by corresponding kits (Beijing Huaxia Ocean Technology Co., Ltd. Beijing, China) according to the manufacturer's instructions.

### 2.5 Quantitative PCR assay

The total RNA in the liver samples was extracted by using TRIzol according to the manufacturer's instructions. The purity of the extracted RNA was detected by using an ultra-micro spectrophotometer (BioDrop, Cambridge, UK). Hi Fi-Script First Strand cDNA Synthesis Kit was used to prepare cDNA. The relative expression of *Pla2g15* and *gad1* mRNA was performed by quantitative PCR assay using the Bio-Rad CFX96 Real-Time System (Bio-Rad, Hercules, CA). The primers used in this study are listed in Supplementary Table S1 (*Gapdh* as housekeeping gene). The relative transcription level was calculated using the 2^−Δ*ΔCt*^ method.

### 2.6 Diversity analysis of the gut microbiota

The feces samples in different groups were collected and frozen at −80°C, put in dry ice and sent to Hangzhou Guhe Health Co., Ltd. (Hangzhou, China) for high-through sequencing of the 16S rRNA genes with the Illumina HiSeq platform. The amplification region is V3-V4 region. The reads were assigned with >97% identity to bacteria by using the Bayesian algorithm from the Ribosomal Database Project (RDP). Operational taxonomic units (OTUs) were classified using the Silva_111 16S rRNA database (13).

### 2.7 Statistical analysis

All data are presented as the mean ± standard deviation (x ± SD). The significance between the two groups of samples was tested using a *T*-test. The significance between the means of three or more sample groups is analyzed using One-way ANOVA. All significance analyses were performed with GraphPad Prism 8 (RRID: SCR_002798. ^*^p < 0.05, ^**^p < 0.01, ^***^p < 0.001.

## 3 Results

### 3.1 W112 alleviated depressive behavior in CUMS mice

**Figure 2:**
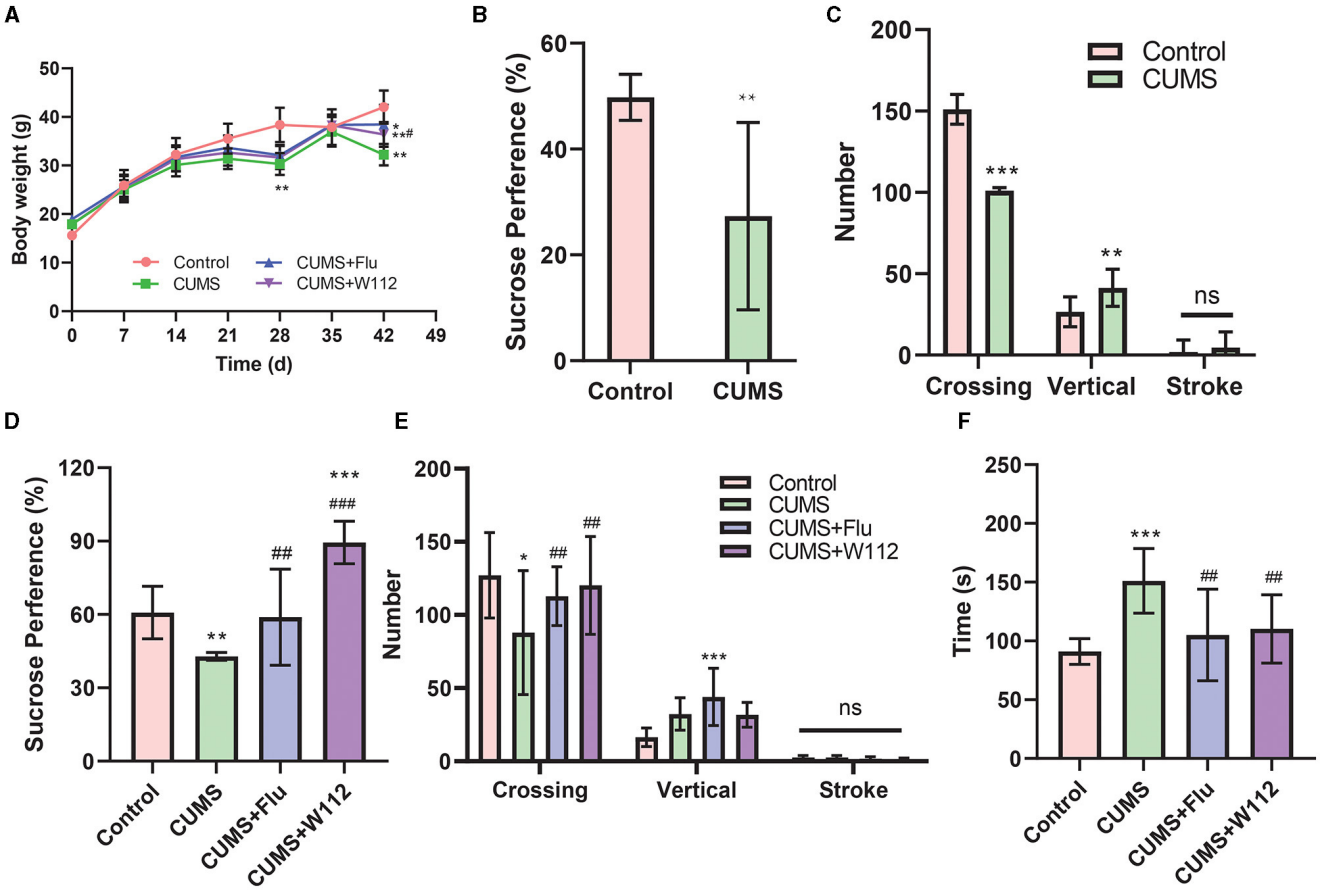
Behavioral evaluation. **(A)** Mice body weight. **(B)** Sucrose preference rate on 28th d. **(C)** The number of crossing, vertical and stroke on 28th d. **(D)** Sucrose preference rate on 42nd d. **(E)** The number of crossings, verticals, and strokes on 42nd d. **(F)** The immobility time of FST on 42nd d. *Compared with control, *p < 0.05, **p < 0.01, ***p < 0.001; ^#^compared with CUMS, ^#^*p* < 0.05, ^##^*p* < 0.01, ^###^*p* < 0.001. ns, not significant. *n* = 10.

Compared with the mice in control group, the mice in CUMS groups showed a decrease in body weight on the 28th day (38.34 ± 3.55 g vs. 30.32 ± 2.72 g, *p* < 0.01; Figure 2A) and sucrose preference (49.77 ± 4.33 vs. 27.34 ± 17.7, *p* < 0.01; Figure 2B). The results of OFT on the 28th day showed that 4-weeks of chronic stress induced the alteration of behaviors in mice. The mice in CUMS groups showed a decrease in the number of crossing (151.07 ± 9.17 vs. 101.02 ± 1.86, *p* < 0.001; Figure 2C) and an increase in the number of verticals and strokes (Figure 2C). It was preliminarily considered that the CUMS modeling was successful according to the anhedonia, exercise activity reduction and the tension status showed in the behavior test.

The effect of W112 on depression status of the CUMS mice was evaluated on the 42nd day. Compared with the CUMS group, the body weight in the CUMS+W112 group and CUMS+Flu group was increased (32.24 ± 2.20 g vs. 36.38 ± 2.56 g, *p* < 0.05). At the same time, all behavioral abnormalities in CUMS mice were reversed by the supplement of W112. Compared with the CUMS group, significantly increase in sucrose preference (42.86 ± 1.56 vs. 89.45 ± 8.73, *p* < 0.01) and number of crossing (87.88 ± 42.35 vs. 120.18 ± 33.35) and decrease in the immobility time (151.17 ± 27.43 vs. 110.29 ± 29.08, *p* < 0.01) were observed in CUMS+W112 group (Figures 2D–F). From the above results, it can be seen that supplement of W112 for 2 weeks could alleviate depression symptoms in CUMS mice.

### 3.2 W112 regulated the concentrations of depression-related biomarkers in CUMS mice

The biomarkers related to 5-HT metabolism in hypothalamic, Trp metabolism in serum and the hypothalamic–pituitary–adrenal (HPA) axis-related biomarkers were measured to determine the effect of W112 on CUMS mice.

**Figure 3:**
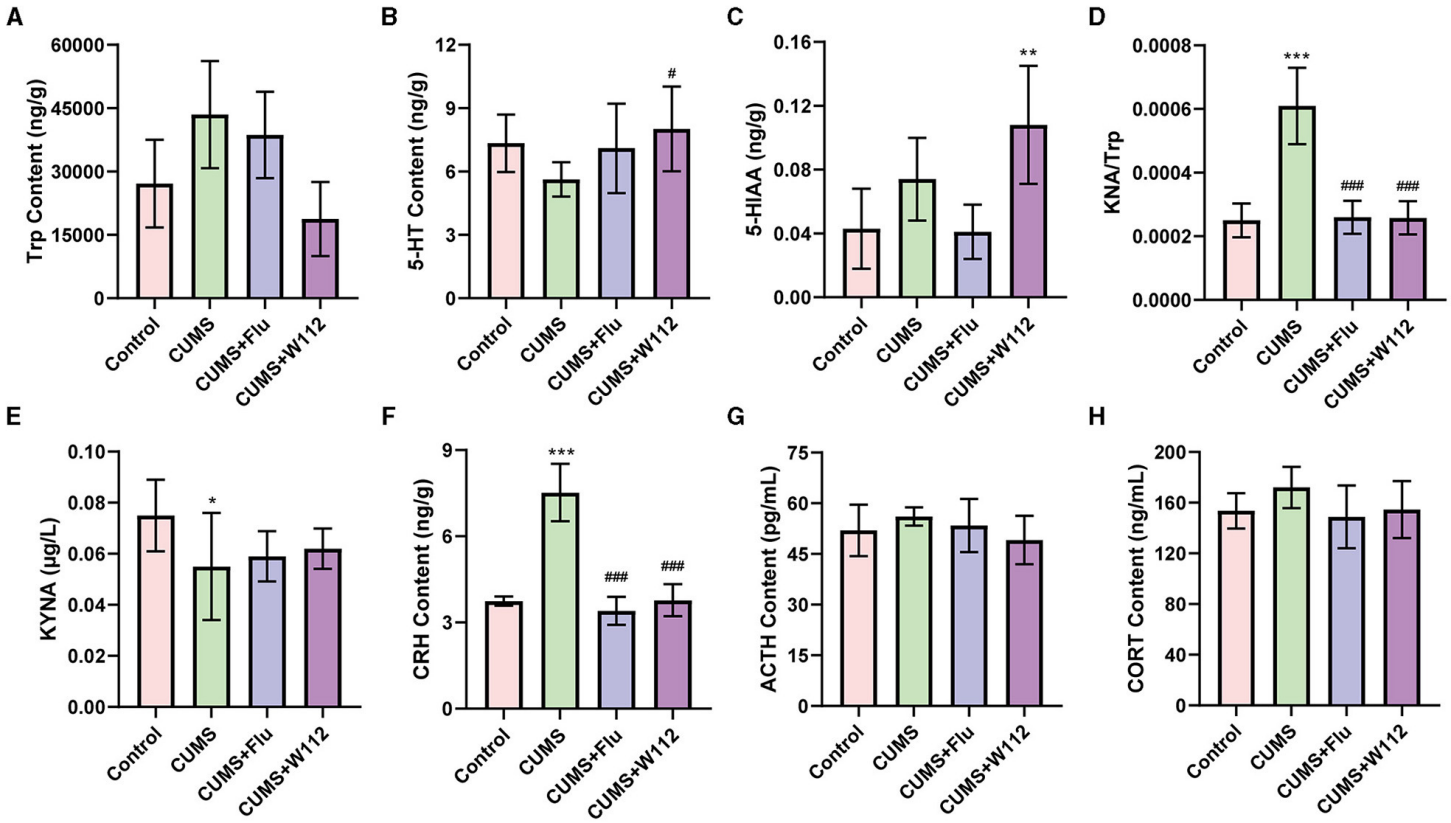
Contents of biomarkers related to depression in each group of mic. **(A)** Tryptophan (Trp) content in hypothalamus. **(B)** 5-hydroxytryptamine (5-HT) content in hypothalamus. **(C)** 5-hydroxyindoleacetic acid (5-HIAA) content in hypothalamus. **(D)** Kynurenine (KYN)/Trp ratio in serum. **(E)** Kynurenic acid (KYNA) content in serum. **(F)** Corticotrophin releasing hormone (CRH) content in hypothalamus. **(G)** Adrenergic hormone (ACTH) content in serum. **(H)** Corticosterone (CORT) content in serum. *Compared with control, *p < 0.05, **p < 0.01, ***p < 0.001; ^#^compared with CUMS, ^#^*p* < 0.05, ^###^*p* < 0.001. *n* = 10.

For the 5-HT metabolism in the hypothalamus, the contents of Trp, 5-HT, and 5-HIAA in the hypothalamus of mice were detected. Trp is an essential amino acid for the synthesis of 5-HT in 5-HT metabolism. 5-HIAA is the final product of 5-HT catabolism. The content of Trp showed no significant difference among all groups (Figure 3A). However, in the CUMS group, the content of 5-HT was significant decreased compared with the control group. Meanwhile, the content of 5-HT in the hypothalamus of the CUMS+W112 group was significantly increased (5.62 ± 0.81 vs. 8.02 ± 2.01 ng/g, *p* < 0.05; Figure 3B). Besides, among the four groups, the content of 5-HIAA was highest in the CUMS+W112 group (Figure 3C).

For the Trp metabolism in the serum, the contents of KYN and KYNA in the serum of mice were detected. The KYN/Trp, which represented a disorder in Trp metabolism of CUMS mice, was calculated. The results showed that, compared with the control group, the KYN/Trp was significantly increased in the CUMS group [(2.50 ± 0.53) × 10^−4^ vs. (6.1 ± 1.2) × 10^−4^, *p* < 0.001]. Compared with the CUMS group, the KYN/Trp in the serum was significantly decreased in the CUMS+W112 group and CUMS+Flu group [(6.1 ± 1.2) × 10^−4^ vs. (2.58 ± 0.52) × 10^−4^, *p* < 0.001; Figure 3D]. The content of KYNA, which is a neuroprotective product of Trp metabolism, was significantly decreased (0.075 ± 0.014 vs. 0.055 ± 0.021 μg/L, *p* < 0.05) in the CUMS group compared with the control group (Figure 3E). Compared with the CUMS group, the KYNA content showed an upward trend in the CUMS+W112 group and CUMS+Flu group (Figure 3E). The above results showed that W112 significantly improved the damage of Trp metabolism pathway in the serum caused by CUMS.

The CRH content in the hypothalamus and the ACTH and CORT content in the serum were detected to evaluate the function of HPA axis. As shown in Figure 3F, compared with the control group, the CRH showed an excessive secretion in the CUMS group (3.74 ± 0.16 vs. 7.52 ± 1.01 ng/g, *p* < 0.001). The CRH content was significantly reduced in CUMS mice administration with W112 or Flu (7.52 ± 1.01 vs. 3.77 ± 0.56 ng/g, *p* < 0.001). Additionally, the content of ACTH and CORT in serum were not significantly different among the groups (*p* > 0.05; Figures 3G, H).

### 3.3 W112 modulated the gut microbiota in CUMS mice

**Figure 4:**
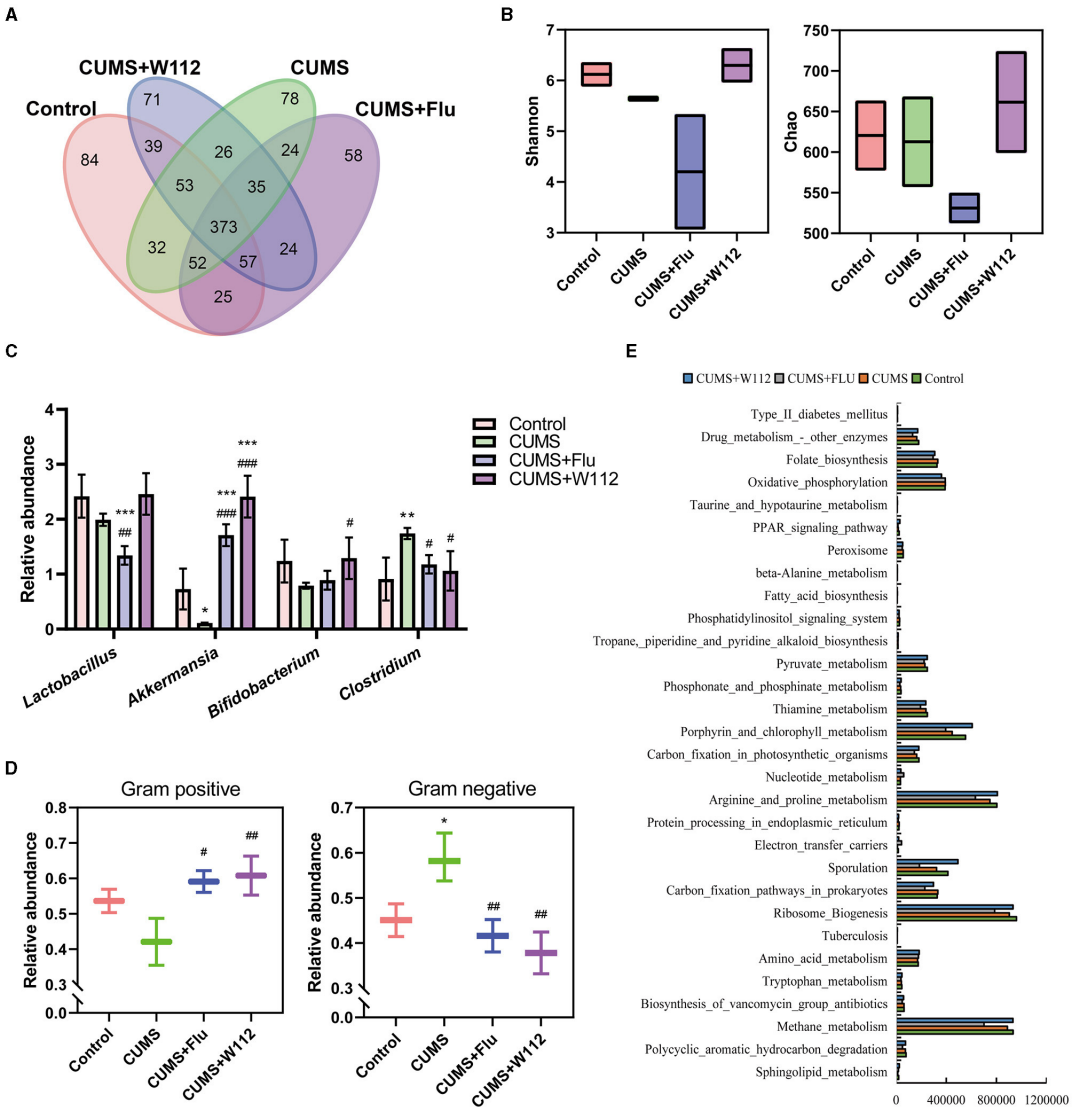
Analysis of gut microbiota of mice in each group. **(A)** Venn diagram at the OTU level (overlapping parts represent common OTU, non-overlapping parts represent unique OTU, and numbers represent the number of OTU). **(B)** Alpha diversity analysis (Left: Shannon index, Right: Chao index). **(C)** The relative abundance of *Bifidobacterium, Lactobacillus, Akkermansia*, and *Clostridium* in each group of mice. **(D)** The relative abundance of Gram positive bacterium and Gram negative bacterium in each group of mice. **(E)** Histogram of the top 30 abundant metabolic pathway genes (the horizontal axis is the sample number, and the vertical axis is the metabolic pathway). *Compared with control, *p < 0.05, **p < 0.01, ***p < 0.001; ^#^compared with CUMS, ^#^*p* < 0.05, ^##^*p* < 0.01, ^###^*p* < 0.001. *n* = 10.

In view of the crucial role of gut microbiota in gut-brain axis, the effect of W112 on gut microbiota in CUMS mice was investigated by using 16S rDNA high-throughput sequencing. As shown in Figure 4A, the number of same OTUs between the CUMS+W112 group and the control group was 522, while only 487 between the CUMS+W112 group and CUMS group. It meant that oral administration of W112 alter the composition of the gut microbiota in CUMS mice similar to the normal mice. For the α diversity, it was found that the Chao index and Shannon index in the gut microbiota were increased in the CUMS+W112 group compared with the other groups (Figure 4B). Subsequently, the gut microbiota was annotated at the genus level. It can be seen from Figure 4C, compared with the CUMS group, CUMS+W112 group showed an increased in the relative abundance of beneficial bacteria (*Lactobacillus, Akkermansia*, and *Bifidobacterium*) and a decreased in the relative abundance of harmful bacteria (*Clostridium*). Then, the phenotype of intestinal microorganisms was predicted by BugBase. It can be seen from Figure 4D, compared with the CUMS group, the relative abundance of gram positive bacteria was increased in the CUMS+W112 group. For the relative abundance of gram negative bacteria, CUMS group showed an significantly increased compared with the other groups. Finally, metabolic function of the gut microbiota was predicted by PICRUSt from the from the taxonomic information. As shown in Figure 4E, the genes in the Trp metabolic pathway have the top 30 relative abundance. The results showed that the abundance of genes related to Trp metabolism in the gut may lead to the changes in tryptophan metabolism in mice.

### 3.4 W112 relieved liver injury caused by CUMS

**Figure 5:**
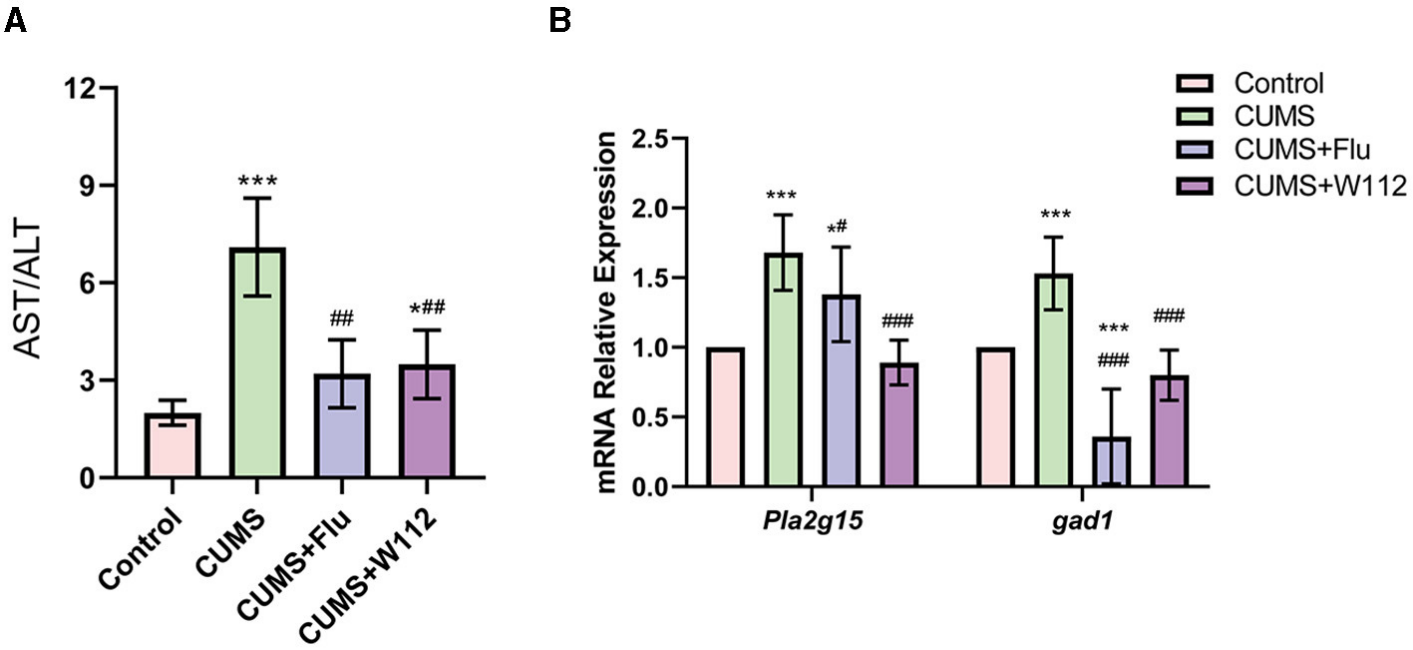
Effect of W112 on liver injury. **(A)** AST/ALT level of mice in each group. **(B)** The mRNA expression levels of *Pl2g15, gad1* in the liver of mice in each group. *Compared with control, *p < 0.05, ***p < 0.001; ^#^compared with CUMS, ^#^*p* < 0.05, ^##^*p* < 0.01, ^###^*p* < 0.001. *n* = 10.

It is reported that the imbalanced gut microbiota in depression mice would increase the content of intestinal LPS, which would disrupt the intestinal barrier and enter the liver to cause liver injury. To further determine the effect of W112 on CUMS-induced liver injury, the activities of ALT and AST in serum and the expression of injury-related genes in the liver were examined. It can be seen that the AST/ALT ratio was increased in the CUMS group compared with the control group (1.99 ± 0.38 vs. 7.09 ± 1.50, *p* < 0.001). Meanwhile, the relative transcription level of *Pla2g15* and *gad1*, which related to phospholipid and bile acid metabolism respectively, were both increased in the CUMS group (Figures 5A, B). After supplemented with W112 for 2 weeks, compared with CUMS group, the AST/ALT ratio of the serum and the relative transcription level of *Pla2g15* and *gad1* showed significantly decrease (*p* < 0.001) in the CUMS+W112 group (Figures 5A, B). The above results indicated that W112 significantly alleviated the liver injury caused by CUMS.

## 4 Discussion

Depression is a chronic and easily recurrent emotional handicap disease, affecting the daily life of individuals in severe cases. In recent years, it has been recognized that probiotics, especially *Bifidobacteria*, could alleviate depression by modulating the gut microbiota. A clinical study confirmed that depressive mood was improved by replenishing *Bifidobacterium* spp. and improving the dietary intake (14). However, the research on the antidepressant effect of probiotics mainly focused on the improvement of central nervous system abnormalities, while ignoring the role of other organs (such as liver) in the improvement process of probiotics. In this study, the effect of a novel *Bifidobacterium pseudocatenulatum* W112 on the depression behavior in CUMS mice was investigated from the perspective of alleviating effect on the liver injury.

According to the Diagnostic and Statistical Manual of Mental Disorders (5th ed) issued by the American Psychiatric Association in 2013 (15), depression have about 1,000 kind of symptoms (16) and could be classified to 19 subtypes (17). Among the numerous symptoms, depressive mood and/or anorexia should be included for the diagnosis of depression. Therefore, the depression-like behaviors, which mainly manifesting anxiety and depression, were considered to be important for the diagnosis of depression. In animal studies, the depression-like behaviors could be assessed through behavioral experiments such as SPT, OFT, and FST. SPT, OFT, and FST are often used to assess anorexia, autonomy and inquiry behavior in strange environments, desperate behavior, respectively. In this work, CUMS mice showed the anhedonia, exercise activity reduction and the tension status in the behavior test. While, these symptoms were improved after oral administration of W112 (Figure 2).

The normalization of neuroendocrine activity is considered an important criterion for determination of antidepressant function (18). Depression is usually accompanied by the abnormal function in the HPA axis (19) and a decrease in 5-HT levels in the brain (20). When under stress, the hypothalamus would excessively release CRH to induce the release of ACTH in pituitary, which thereby causes the CORT release by adrenal cortex (21). The decreased CRH content after W112 administration in CUMS mice indicated that oral administration of strain W112 could reverse the HPA axis hyperactivity in depression (Figure 3F). The 5-HT in the central nervous system (CNS) is another key neurotransmitter for the regulation of emotions. The decreased content of 5-HT in CUMS mice was restored in the hypothalamus by the intervention of W112 (Figure 3B). Based on the above results, it can be concluded that W112 can promote the normalization of neuroendocrine activity by regulating the content of relevant biomarkers in CUMS mice, which indicated that W112 has an antidepressant function.

In recent years, increasing evidences pointed out that imbalance of gut microbiota has become an important pathological reason for depression (22). Animal tests also clarified the differences of the gut microbiota in various types of depressed animal models, including bilateral olfactory bulb excision models (23), maternal separation models (24), social division models (25), chronic variable stress model (26) and chronic restraint stress model (27). In this study, the analysis of gut microbiota abundance in CUMS mice also found that compared with the normal group, the abundance of *Bacteroidetes* and *Clostridium* increased while the abundance of *Lactobacillus* and *Bifidobacteriu* decreased. It is noteworthy that the intervention of W112 significantly increased the abundance of *Lactobacillus* and *Bifidobacterium* in the CUMS mice. Importantly, the abundance of *Akkermansia*, an important gut member, was also increased in CUMS mice after supplementation with W112 (Figure 4C). It has been proven that *Akkermansia* could improve the immunity status by acting on the function of the intestinal barrier, including acting on the TLR2 receptor to restore the intestinal barriers, maintaining the intestinal mucosa integrity and, reducing the intestinal LPS content (28–30). Therefore, the anti-depression effect of strain W112 might be achieved by its modulation effect on the composition of gut microbiota in CUMS mice.

The prediction of gut microbiota phenotype showed that W112 reduced the relative abundance of gram negative bacteria in CUMS mice. Since the excessive LPS produced by the intestinal gram negative bacteria may destroy the intestinal barrier, W112 reduced the risk of intestinal barrier destruction. What's more, the study has suggested that excessive intestinal LPS may cause liver injury through the portal vein circulation (31). In this work, CUMS mice showed a significantly increased AST/ALT, which usually represents the liver injury in clinical. While, the AST/ALT of CUMS mice decreased after supplemented with W112, which indicated that the CUMS induced liver injury was improved. Additionally, modern pharmacological studies suggest that chronic stress might induce liver injury by interfering with hepatic function (9), hepatic metabolic profile (32), and the genes expression in phospholipid and primary bile acid biosynthesis pathways (33). To further clarify the mechanism of W112 in improving liver injury, the *pla2g15* and *gad1* genes were selected to evaluating the phospholipid metabolism (34) and bile acid (BA) synthesis (35) of liver, respectively. The results showed that W112 could alleviate CUMS induced liver injury by regulating phospholipid metabolism and bile acid metabolism.

In summary, W112 could significantly improve the depressive and liver injury symptoms caused by CUMS based on the gut-brain axis and the gut-liver axis respectively through modulating gut microbiota. Therefore, the gut-liver-brain axis is a potential connecting pathway between the antidepressant effects of W112 and its alleviation of liver injury.

## Data Availability

The datasets presented in this study can be found in online repositories. The names of the repository/repositories and accession number(s) can be found at: https://www.ncbi.nlm.nih.gov/, PRJNA1134429.
