# Supplementary material for: Bifidobacterium pseudonumeratum W112 alleviated depressive and liver injury symptoms induced by chronic unpredictable mild stress via gut-liver-brain axis
Source: Front Nutr. 2024 Aug 19;11:1421007. doi: 10.3389/fnut.2024.1421007 (PMC11366711; doi:10.3389/fnut.2024.1421007)
Supplement: Supplementary file 1 [file Table_1.DOCX]

Supplementary Material

# Methods

## Behavioral Test

Using body weight measurement, sucrose preference experiment, open field experiment, forced swimming experiment to evaluate the behavior of mice before and after CUMS and after intervention.

## The detail protocol of sucrose preference test (SPT)

SPT is often used to assess anorexia. Mice were adapted to consume sucrose water for 24 h, and were deprived of water and food for 24 h. After that, they were treated with 1 bottle of 1% sucrose solution and 1 bottle of ordinary water for 12 hours; the bottle position was rotated after 6 hours to avoid side preferences [1]. The consumption of sucrose solution and ordinary water was measured by weight, and the sucrose preference (SP) value is calculated by the following formula: SP=Sucrose consumption/(Sucrose consumption+Water consumption)×100%.

## The detail protocol of open field test (OFT)

The OFT was described by Yen-Wenn Liu [2]. It can reflect the autonomy, inquiry behavior and stress of experimental animals in strange environments. The number of mise crossing can reflect motor activity, the number of vertical can reflect autonomous exploration ability, and the number of strokes can represent tension. OFT need to be performed in a quiet, low-light environment. Animals were placed in black open boxes with length, width, and height of 80, 80, and 40 (cm). The bottom surface is divided into 25 equal-sized squares with a side length of 16 cm by a yellow paint line. The measurement was started after the animals were placed in the center of the grid, and 5 minutes each time. The number of crossings, verticals and strokes per unit time were recorded. After the end of each animal experiment, use 75% alcohol to remove the smell left by the animal before starting the next animal experiment. Analysis of various behavioral indicators by double-blind method.

## The detail protocol of Forced swimming test (FST)

The FST is used to assess desperate behavior and is one of the most widely method in antidepressant behavior [3]. Each mise was placed in a large cylinder (18 × 40 cm) filled with water and forced to swim for a total of 6 minutes; the immobility time was recorded during the last 4 minutes. (Water temperature: 25 ℃, water depth 23cm). Immobility is defined as keeping floating in the water with only tiny movements to keep the head above the water.

# Reference

1 Garza JC, Guo M, Zhang W, Lu XY (2012) Leptin restores adult hippocampal neurogenesis in a chronic unpredictable stress model of depression and reverses glucocorticoid-induced inhibition of GSK-3β/β-catenin signaling. Mol Psychiatry. 17(8):790-808. doi: 10.1038/mp.2011.161.

2 Liu YW, Liu WH, Wu CC, Juan YC, Wu YC, Tsai HP, Wang S, Tsai YC (2016) Psychotropic effects of Lactobacillus plantarum PS128 in early life-stressed and naïve adult mice. Brain Res. 1631:1-12. doi: 10.1016/j.brainres.2015.11.018.

3 Zorrilla EP, Koob GF (2010) Animal models of depression. Drug Discov Today. 15(9-10):371-83. doi: 10.1016/j.drudis.2010.02.011.

# Supplementary Tables

**Table S1** RT-qPCR primers and main features

| Name | Primers（ 5’-3’） | length | Sequence size（bp） | Tm（℃） | GC% |
| --- | --- | --- | --- | --- | --- |
| *Pla2g15* | F:TGAATGGGTTTTGGTTTGCG  R:AAGCCCCTAGCTGGGACTCTAA | 20  22 | 89 | 61.7  61.5 | 45  54.5 |
| *gad1* | F: GGCACGACTGTTTATGGAGCG  R: GGTGACTGAATTGGCCCTTTCT | 21  22 | 165 | 63.0  62.1 | 57.1  50.0 |
| *Gapdh* | F:ATGATTCTACCCACGGCAAG  R:CTGGAAGATGGTGATGGGTT | 20  20 | 89 | 61.6  62.4 | 53.1  51.0 |
